# Finding your scientific story by writing backwards

**DOI:** 10.1007/s42995-021-00120-z

**Published:** 2021-10-13

**Authors:** David J. S. Montagnes, E. Ian Montagnes, Zhou Yang

**Affiliations:** 1 Department of Evolution, Ecology, and Behaviour, University of Liverpool, BioSciences Building, Liverpool, L69 7ZB UK; 2 Private Office, 31 Baldwin Street, Port Hope, ON L1A 1S3 Canada; 3 School of Biological Sciences, Nanjing Normal University, Nanjing, 210023 China

**Keywords:** Scientific narrative, Scientific pedagogy, Scientific writing, Story-telling, Writing structure

## Abstract

To succeed, a scientist must write well. Substantial guidance exists on writing papers that follow the classic Introduction, Methods, Results, and Discussion (IMRaD) structure. Here, we fill a critical gap in this pedagogical canon. We offer guidance on developing a good scientific story*.* This valuable—yet often poorly achieved—skill can increase the impact of a study and its likelihood of acceptance. A scientific story goes beyond presenting information. It is a cohesive narrative that engages the reader by presenting and solving a problem, with a beginning, middle, and end. To create this narrative structure, we urge writers to consider starting at the end of their study, starting with writing their main conclusions, which provide the basis of the Discussion, and then work backwards: Results → Methods → refine the Discussion → Introduction → Abstract → Title. In this brief and informal editorial, we offer guidance to a wide audience, ranging from upper-level undergraduates (who have just conducted their first research project) to senior scientists (who may benefit from re-thinking their approach to writing). To do so, we provide specific instruction, examples, and a guide to the literature on how to “write backwards”, linking scientific storytelling to the IMRaD structure.

## Publish or perish

**Fig. 1:**
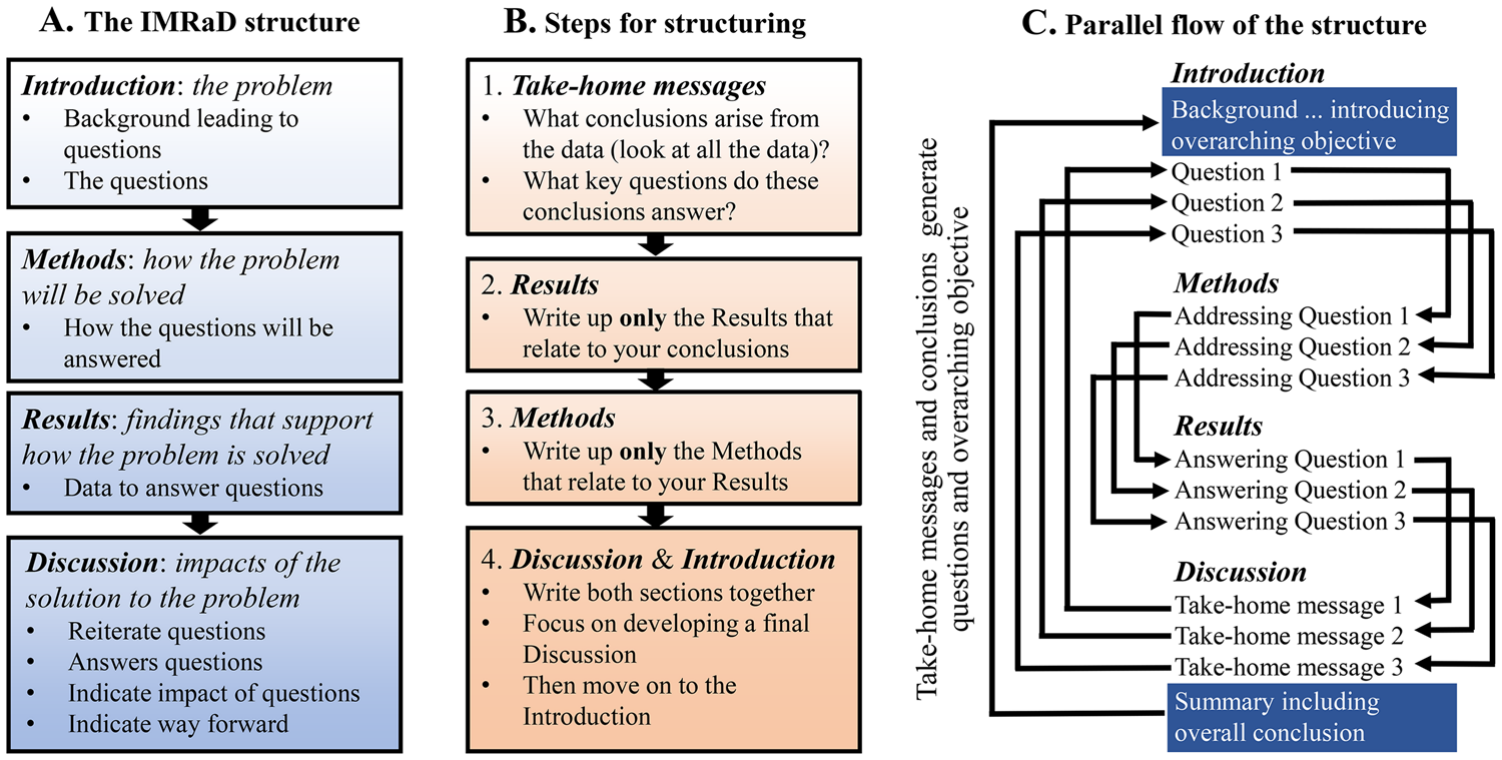
The scientific story. **A** The IMRaD structure of a scientific story; **B** Our suggested steps for developing a scientific story; **C** Parallel structure and the flow of the scientific story. B. and C. are outlined in detail in the section “Steps towards developing a scientific story”

Writing well is an essential skill in science. Many resources offer guidance on producing concise, efficient, and convincing papers (Table 1), which are mostly based on the classic Introduction, Methods, Results, and Discussion (IMRaD) structure (Fig. 1A). For general rules on writing we suggest sources presented in Table 1. Here, we focus on an important aspect of writing often overlooked in these resources: developing the *scientific story.* Embracing this valuable skill—one that underlies any good paper—can increase the impact of your work and the likelihood of it being accepted in highly rated journals (Turabian 2019).

**Table 1:** A selection of sources for structuring a scientific paper, presented by date/authors, with no prejudice. These are mostly recent books that we see as useful sources. By no means is this an exhaustive list; many other good books and articles are available on the subject. We encourage authors to find sources that best suit their needs. Prices are for paperbacks (when possible) and are only an approximate value, rounded to the nearest £5, and determined in 2021. Some sources may also come as less expensive e-books. Furthermore, if you are on a budget, older editions of many of these and other books are often as useful as the newest edition, and might be significantly less expensive

Tile	Authors/date/ISBN	Notes/cost
Editing and publication	Montagnes (1988) ISBN (none)	A simple, practical guide to effective writing, designed for editors at research institutes but equally useful for authors. Free on-line as a pdf
Writing science: how to write papers that get cited and proposals that get funded	Schimel (2012) ISBN: 978-0-19-976024-4	A comprehensive coverage of the subject, with useful insights into “story telling” £25
Scientific style and format: The CSE manual for authors, editors, and publishers, 8th edn	Council of Science Editors (2014) ISBN: 9780226116495	A definitive style guide, and an invaluable resource for authors, but not a useful “how-to” book. £55
*Guidelines for scientific paper writing (Written in Chinese)	Zhao and Ding (2014) ISBN: 9787030415455	Introduces what to do at each step and how to build the relationship between parts. Aimed at students and new researchers. £5 (in China)
How to write and publish a scientific paper	Gastel and Day (2016) ISBN: 9781440842801	In its 8th edition, this is an excellent all-round resourced. We recommend it to students. £30
*English for writing research papers 2nd edn	Wallwork (2016) ISBN: 9783319260921	A thorough training book, from sentence to manuscript structure. For non-English speakers but useful for all, from beginners to mature writers. £20
How to write a good scientific paper	Mack (2018) ISBN 9781510619135	A useful general guide, with good guidance on figures. £30
Writing and publishing a scientific research paper	Parija and Vikram (2018) ISBN: 9789811352119	A useful general guide, but expensive. £70
How to write a scientific paper: an academic self-help guide for PhD students	Saramäk (2018) ISBN: 9781730784163	Includes parallels to our writing backwards approach and a useful general guide. Offers guidance on finding main ideas from your work (i.e., the take-home messages). £10
*Science research writing: for native and non-native speakers of English 2nd edn	Glasman-Deal (2020) ISBN: 9781786347848	A useful training book, for researchers at the start of their career. £20
Scientific writing = thinking in words	Lindsay (2020) ISBN: 9780643100466	Short and easy to read, and full of useful information for all career stages. £15

*Books that focus on helping non-English speakers

### The scientific story

**Fig. 2:**
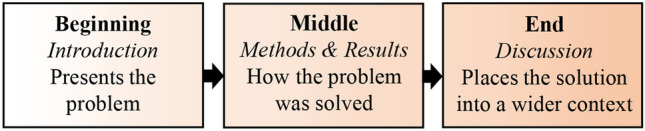
The scientific story seen as three parts, mapping on to the IMRaD structure (Fig. 1A)

Story-telling is part of being human. Stories are an integral part of our lives, from newspapers and novels to blogs and movies. This is because stories have evolved with us as an effective form of communication, including in science (Angler 2020; Clemens 2018; Sanes 2019). But what do we mean by a scientific story? A scientific story goes beyond just presenting information; it is a narrative that uses information (e.g., data) to solve a problem, engaging the reader with both your observations and an appreciation of their impact. The scientific story has a *beginning*, a *middle,* and an *end* (Fig. 2). These three components can, and should, map onto the typical IMRaD structure (Fig. 1A). However, as editors we see many manuscripts that follow the IMRaD structure but do not tell a good scientific story, even when the underlying data clearly can provide one. For example, many studies present the findings without any synthesis or an effort to place them into a wider context. This limits the reader’s ability to gain knowledge and understanding, hence reducing the papers impact. Here, we offer guidance on how to tell your story.

Three structural rules underpin all writing. These rules can be directly applied to developing your scientific story: Rule 1—consider your *audience* (i.e., scientists); Rule 2—consider your *venue* (i.e., scientific journals); and Rule 3—consider your *purpose*. The purpose of scientific research is to collect and analyse data to determine underlying truths and gain understanding through hypothesis testing or the exploration of large data sets. For a thought-provoking review of scientific approaches see Voit (2019). Fretwell (1975) provides philosophical insight into this process.

*“Scientists are responsible for truth, knowledge, wisdom, and understanding. Truth is what is—it is the underlying reality of all existence. Knowledge is what we think we know about truth. Knowledge, however, is always an imperfect assessment, and is always subject to revision and improvement. The realization that there are discrepancies and weaknesses in knowledge is wisdom. Wisdom leads to a process, called the philosophy of science, through which knowledge is modified to better fit the truth...”*

Fretwell (1975) then extended this philosophy to applied science, which is very much one aim of this journal, *Marine Life Sciences & Technology* (MLST).

*“We may think of understanding as what we use in order to adequately apply our wisdom and our knowledge in guiding our actions. While applied scientists seek understanding, basic scientists seek knowledge.”*

As scientists, regardless of how we find and apply our answers, the order of our writing is generally expected to follow the IMRaD structure (Fig. 1A). We argue, however, that trying to write a manuscript following this structure will often impede developing a good story (Fig. 2). Instead, we suggest that authors should consider writing backwards (Fig. 1B; Magnusson 1996; Sanes 2019). In the next sections we outline this approach.

### Writing backwards?

**Fig. 3:**
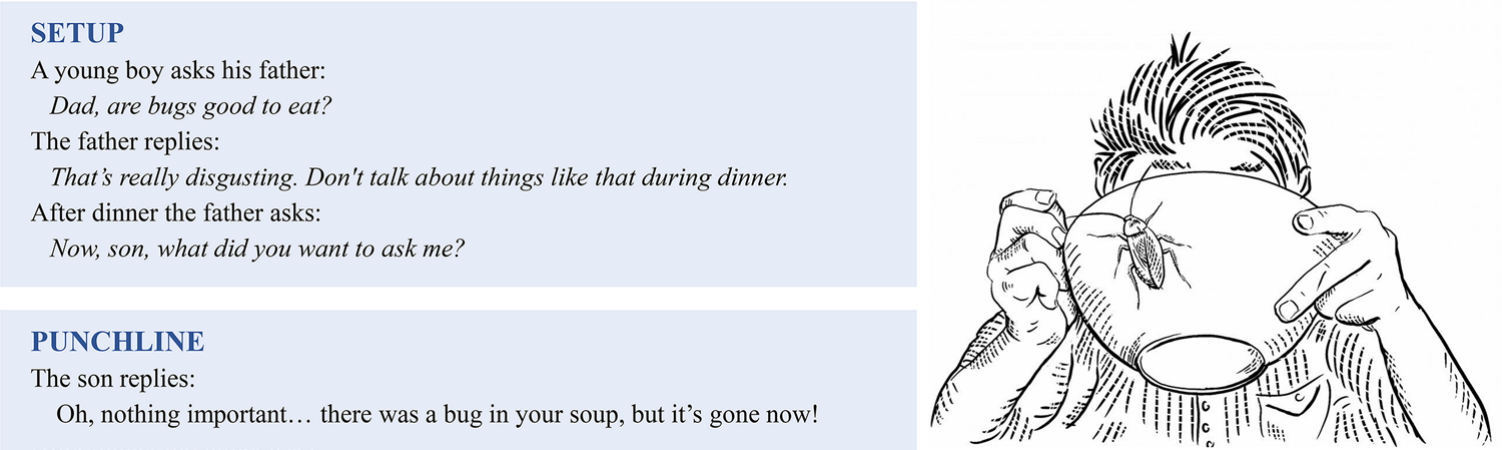
An example of a joke with the setup and punchline, in this sense following the same structure as a scientific story (Fig. 1)

Writing backwards may seem like an odd concept, but it’s not. Think about telling a joke to your friends. Knowing the punchline is essential. You build up to it, and the punchline makes the joke. Of course, a good setup to the punchline is also crucial, but without a perfect conclusion, the joke won’t work (Jodłowiec 1991). In fact, many comedians start writing their jokes with a punchline in mind—or at least a rough version of it—and then craft the setup (Fig. 3). In other words, the joke is constructed backwards from the punchline, even though that’s not how you tell it. A scientific story is no different.

## Steps towards developing a scientific story

### Step 1: where to begin?

#### Step 1.1: the punchline

What then is the first step? It is not to *write* the Introduction, the Methods, or the Results—although you will, undoubtedly, have made extensive notes on all of these sections, including producing working versions of figures and tables that will reveal trends and suggest outcomes. Rather, the first step in writing backwards is to decide what your main conclusions are, often called the *take-home messages*. These are the exciting and novel ideas, trends, and concepts that will arise from the critical appraisal of your data. They are the messages that your reader will remember, or “take home” with them, after reading your paper (Fig. 1B, *Step 1*). The take-home messages will dictate the structure of your entire story and will lead to an overall summary (Fig. 1C). This is your punchline!

The purpose of your study should revolve around these take-home messages. They will, almost certainly, require considerable time and broad thinking to develop. This is the intellectual part of your research that precedes writing. If you are lucky, or maybe better to say if you have planned well, you will have anticipated the take-home messages, based on your carefully crafted proposal. However, more often than not, unexpected results arise—especially from biological experiments—and we must be open to them (Voit 2019). Then, the take-home messages will arise from analysing your data, and your overall conclusions will be a synthesis of the take-home messages (but please read *A cautionary note,* below). While it is beyond the scope of this editorial to provide extensive guidance on this first step (which is inevitably study-specific), Box 1 offers guidance on developing take-home messages.

Box 1 Developing* take-home messages* This is the creative, and hopefully enjoyable, part of your study. It is where you take the facts that you’ve worked so hard to obtain and shape them into useful and interesting points. It is not sufficient to just tell the reader *what* you have found. You need to indicate *why* it is important and *how* it can be used. To do this effectively is often a real challenge. Below are some suggestions on how to develop these take-home messages.

1. Scientists are often very visual (Mathewson 2005). If you are also visual, then try printing off all the working-versions of your figures and tables (include everything at this point), laying them out, and using these visual sources to help you recognise the main points and patterns of your story.
2. You could produce a “storyboard”. For example, use a computer and possibly a digital projector to facilitate group discussions (see https://bcourses.berkeley.edu/courses/1492853/pages/tip-of-the-week-use-powerpoint-to-storyboard-your-paper).
3. Tell your story to anyone who will listen*. This could include peers, students, and even family, and could occur at scientific meetings, lab meetings, or around the dinner table. It is surprising what insights arise as you try to simplify your study and tell someone who is not familiar with your work *what* you have found, *why* it’s important, and *how* it may be used. If you’re able to engage these people they will undoubtedly ask difficult questions that will require you to rethink the way that you tell your story.
  *Clearly, for sensitive material (e.g., that may lead to financial rewards), you must be a little careful who you talk to.
4. *Chance favours only the prepared mind* (Pasteur 1854, https://en.wikiquote.org/wiki/Louis_Pasteur). In the context of storytelling this equates to, *know all of the background information, and you will recognise take-home messages when the data present them*. Make sure that you are well- and widely read on your subject, and beyond!
5. Read papers critically, and look for studies that tell a good story. Look at the structure and flow of papers, and think about which ones do a good job of telling a story and why they do so. Learn from their approaches.
6. We suggest that you try to have no more than three take-home messages in your study. Too many points will confuse your reader, and you will not have impact. Then make sure that all your take-home messages are combined to support your final general conclusion.
7. Try to present your take-home messages and the overall conclusions in one sentence, each. This will focus your mind, requiring you to simplify complex ideas to their important essence.

**Step 1.2: the order**

The next essential step is to decide the order in which to present your take-home messages. If your study only has one message that’s easy, but many studies arrive at more than one message. Try not to have too many, as you will confuse your audience. In fact, if you think that you have several take-home messages, try to condense them into one (or at most three) overarching messages. This will provide focus to your paper, improving its impact (Fig. 1C).

**Fig. 4:**
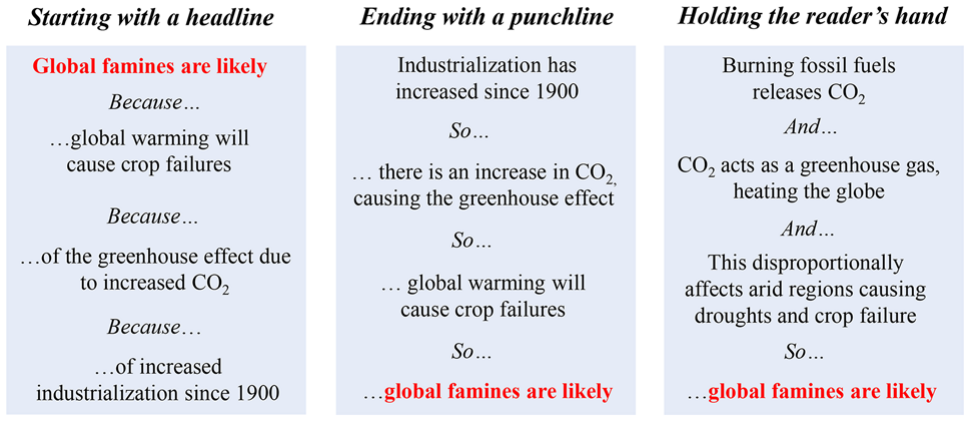
Contrasting approaches for presenting the order of three take-home messages leading to an overall conclusion (in red). In this case, the story is a bit contrived and is imperfect, but hopefully it illustrates the point

The order in which you present the take-home messages is crucial for telling a good story (Angler 2020; Fig. 4). For instance, you may wish to start with your most exciting message, with your purpose being to catch the reader’s attention with a headline concept. Or the narrative might start with the least exciting ideas, ending with a punchline. However, this is often a dangerous approach as you may lose the reader’s attention, which is why we do not encourage using this approach. Another option is that the order may be dictated by a need to provide background concepts first, presenting simple ideas that lead to more complex ones. We call this *“holding your reader’s hand”*, as you guide them through your story. This final structure is often the most sensible to use, as sometimes you need to treat your reader a bit like a child!

Order is essential. There is little more frustrating to an editor or reviewer than reading a manuscript that seems to present ideas haphazardly, requiring the reader to try to put the story together themselves. Do not move on to *Step 2* until you have an order for your structure. Equally, do not be afraid to revise this order later on. There have been times when we have almost finished writing a manuscript, only to rip it up as a better order came to light—back to the drawing board, but ultimately with a much better story! So, at this point do not write the Discussion (wait for *Step 4.1*), but recognise that you now have a good plan for the Discussion, and keep your plan in mind.

Now that you have a plan, you need to think about *parallel structure* as you move backwards. To achieve parallel structure, each section of the IMRaD structure (Fig. 1A) should follow the same pattern in terms of content (Fig. 1C). This order will be dictated by the order of your take-home messages (Fig. 4).

### Step 2: where’s your support?

The next step is to move backwards from the take-home messages and overall conclusions, and formally write up the Results section. Here you should only provide information (i.e., the data and observations) leading to the take-home messages, with no extraneous information to distract the reader. Stick with your plan! Repeatedly ask yourself “Do I need to report these findings to support my take-home messages?” If the answer is “no”, then save that information for another paper, your supplementary material (a potentially good place to add extra stuff; see Pop and Salzberg 2015), or if you really cannot let go of those findings, consider revising your take-home messages to include it (i.e., go back to *Step 1*). At this point you should make your final figures and tables (for guidance see sources in Table 1 and Jambor et al. 2021). Clear and well-structured figures and tables that illustrate your take-home messages are essential for a good story.

### Step 3: how did you get there?

Once you are satisfied with your Results section, move one step further backwards to write the Methods section. Your detailed lab-notes will provide the basis of this section. This process allows you to focus only on the methods used to produce data presented in your Results section. In other words, you can now reduce the extensive records of your methodologies (e.g., your lab book or your initial draft of a Methods section) to only those that relate to your current Results.

Many journals, including MSLT, now place the Methods at the end of the manuscript. This does not mean the Methods are of little consequence. Indeed, the entire study depends on how you obtained your Results. If your methods—both practical and analytical—are inadequate or incomplete, then neither the Results nor the Discussion are worth reading; manuscripts are often rejected solely on the poor methods. In fact, when reviewing papers, we often do not even look at the Results or Discussion if the methods are poor. So, make sure this section adequately outlines how your results were obtained.

### Step 4: fitting it all together

The next step is probably the most challenging: determining a balance between your Introduction and Discussion. Combined, these two sections convince the reader that your study needed to be done and that your take-home messages have impact. The Introduction should be short and to the point. However, sometimes detailed concepts must be presented up-front in the Introduction, allowing the reader to understand the study’s purpose, which can increase the length of the Introduction. The Discussion explains the wider context of your take-home messages, so it can be longer and more speculative than your Introduction (see sources in Table 1 for further guidance on these sections).

At this point do not become fixated on what must be in the Introduction and what must be in the Discussion. As you develop your story large portions of text associated with key concepts may be moved back and forth between the Introduction and Discussion. You may have already drafted a very rough “working Introduction” based on your project’s proposal. If you have, then at this point, it is best to set this draft of the Introduction aside and focus on writing your Discussion. The Introduction will then need further revision after your Discussion is finished because we continue to write backwards.

### Step 4.1 Back to the take-home messages

By now the structure of your Discussion should be fully developed, based on your purpose*,* your take-home messages, your overall conclusions (Fig. 1C), and the carefully considered order in which you plan to present these (Figs. 1C, 4). The Discussion should never simply repeat the Results, nor should it include extensive comparisons to previous findings, unless this was an explicit purpose of your work. Both of these approaches are boring and distracting to the reader. Rather, the Discussion should be a synthesis of your findings and those of others that explores the impact of your take-home messages and their relation to your purpose and overall conclusions (see Table 1 for more guidance). Fortunately, the order that you developed in *Step 1.2* has been well thought out; stick with it and you will not fail!

### Step 4.2: Where are we going?

The Introduction should be the last section that is completed—after all, it is difficult to introduce a topic before it is completed; i.e., until you have fully developed and organised the structure. The Introduction presents the purpose of the study (i.e., the overarching objective and the questions, Fig. 1C) that leads to the take-home messages (remember *Step 1.1*).

One of the most effective means to achieve this goal is to end the Introduction with a clear set of questions that reflect your take-home messages. This provides an overall *problem–solution* structure to the story; i.e., where a problem is raised in the Introduction and a solution is provided in the Discussion (Figs. 1C, 2). These points need not necessarily be phrased as “questions”. They could be “propositions” that will be evaluated, or “hypotheses” that will be tested. Regardless of how they are phrased, these final points will introduce key issues that will be addressed throughout the study.

Ending the Introduction with clear questions/propositions/hypotheses has an added benefit, as the reader can then critically assess whether both the Methods and Results sufficiently address the problem with which the study begins. Given that you have been writing backwards (Fig. 1B, *Steps 1–4*) and have the solutions (the take-home messages), the questions/propositions/hypotheses should naturally arise. For example, for the story presented in Fig. 4 (after providing sufficient background) the author might end the *Introduction* with this paragraph:

*“This study, therefore, examined the impact of climate change on 21st century famine events. To do so, through our literature review and meta-analysis, we addressed the following questions:* (i) *To what extent did industry increase CO*_*2*_* levels in the 20th century?* (ii) *How does CO*_*2*_* alter the greenhouse effect and global warming? and* (iii) *Will warming disproportionately influence arid regions?”*

Clearly, this is a contrived and simplistic example, but it illustrates how questions can be derived from take-home messages. As an aside, following Fig. 1C, if the questions were presented in the above order, then sub-sections within the Methods and Results should have a parallel structure, addressing (*i)*, (*ii)*, and (*iii)*, in the same order, and ending with how the overall conclusions (e.g., relating to the *impact of climate change on twenty-first century famine events)* were obtained.

## A cautionary note

There is one danger in writing backwards, as we and others propose (Magnusson 1996; Sanes 2019). When inappropriately applied, this process can undermine the basic tenets of objective scientific inquiry. By examining the data and determining the take-home messages, we are at least in part ignoring the idea of developing initial (a priori) predictions. Instead, to some extent, we are relying on post hoc (after the fact) observations and interpretations. This post hoc approach is now a recognised and entirely appropriate form of scientific investigation (Voit 2019). Furthermore, our opinion is that all scientific endeavours include some subjectivity and that the crux is the study’s ability to obtain—or at least approach—the truth. In this sense, we emphasise the need for authors to be objective when approaching *Step 1*; i.e., when deciding on the overall conclusions and take-home messages (Fig. 1B, Box 1).

We also caution authors to ensure that the questions/propositions/hypotheses at the end of the Introduction (*Step 4.2*) do not appear too contrived; i.e., they should be general rather than being so detailed that they reflect only the specific findings of the study. There is a fine art to developing a good story. It takes practice and training. Here, and in Table 2, we offer some basic guidance, but we encourage authors to read further so they can develop more nuanced and engaging stories (Table 1).

**Table 2:** Some Do’s and Don’ts when writing your scientific story. This list offers guidance on how to avoid the pitfalls when constructing your story. Much of it is common sense, and we present it to remind the reader that this editorial offers only very directed guidance, some of which may not work for all authors

Do’s	Don’ts
Learn to write all sections in the IMRaD structure (Table 1) before thinking about structuring your story	Ignore the guidelines presented by the journal you are submitting your work to
Consider methods for structuring a paper other than our proposed method of writing backwards	Think that this editorial provides all the answers
Be a good scientist, letting the facts lead to the story	Provide fake news by writing a story based on insubstantial facts, no matter how exciting the story is
Consider all options when interpreting your data and obtaining the take-home messages	Become attracted to one seemingly “sexy” idea, just because it appears to tell a good story
Ensure that you present negative results (i.e., ones that may not support your story), either in the paper or in supplementary material. These can be extremely valuable	Present only positive results (ones that support your story), as this provides non-scientific bias
Use all of your relevant results	Throw away results that disagree with your take-home messages
Collect more data if your take-home messages are unclear	Try to create a story based on poor or insufficient data
Ensure that your data analysis is appropriately targeted to address your questions; i.e., your analysis must lead to the take-home messages	Chose analytical methods that are designed to reveal the trends that you think the story is telling
Provide balanced arguments that consider the breadth of ideas available in the literature	Ignore key references that disagree with the story you have initially developed
When writing the first draft of your paper, try not to worry about making it perfect. You can worry about the grammar and the sentence structure after you get all your ideas in order	Get caught up in the details when writing the outline of the story
After you finish your paper, put it aside for a few days. Then pick it up, with “fresh eyes” and see if the story works	Finish the manuscript and send it off immediately

## Finishing touches

This section provides brief comments on a range of issues that are related to the main points above. Please see them as added advice, and read more widely (e.g., sources in Table 1) if you wish to continue to develop your writing.

### Is it always best to write backward?

**Fig. 5:**
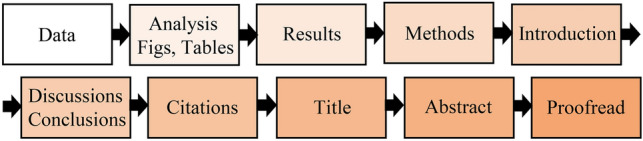
Another possible order for writing a paper, modified primarily from Lippi (2017)

There are many ways to structure a story, just as there are many ways to formulate a joke. The sources in Table 1 present some alternative views, and the articles by Lippi (2017) and Yusoff (2018) offer more specific direction. Both Lippi (2017) and Yusoff (2018) suggest methods similar to our writing backwards approach but follow a slightly different progression (Fig. 5). For instance, in Fig. 5 “Data” and “Analysis” equate to our direction to identify the take-home messages. Both Lippi (2017) and Yusoff (2018) also encourage writing the Introduction before the Discussion. As stated above, we see these two sections as intertwined, but if you have clear take-home-messages, their approach could work better for some authors. Furthermore, contrary to Lippi (2017) (Fig. 5), we would recommend writing the Abstract before writing the Title (although you may have a “working title” the final title should be the last thing you create (see “*Notes on titles and abstracts*”). Finally, some experienced authors start with the Introduction—they argue that writing backwards is not necessary. However, we expect that these experienced authors have in fact written backwards, but done so in their heads—instinctively—not on paper. They have been able to conceive the entire scientific story, before starting the writing process. Most of us, however, are not that smart! We encourage readers to examine these options—and others—to find what works best for them.

### Notes on titles and abstracts

Here we continue to write backwards with the final sections being the Abstract and then the Title. Sources in Table 1 provide guidance on writing both a short, descriptive Title and an informative Abstract (but also see Plakhotnik 2017). Because the Abstract will always appear with the Title, there is no need to repeat the content of the Title within the Abstract.

**Fig. 6:**
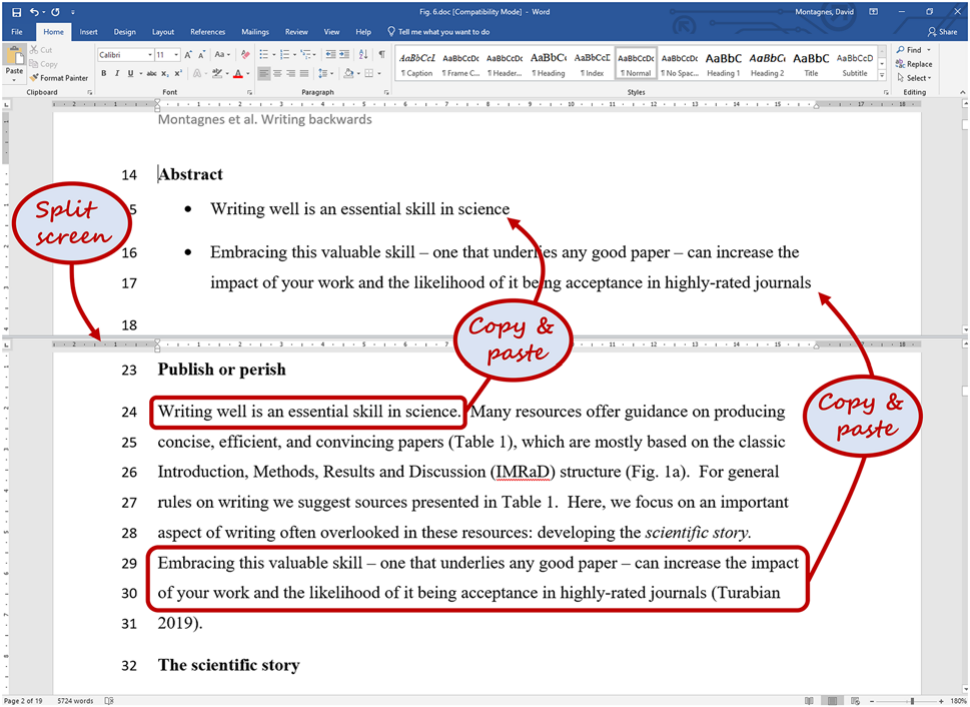
Writing an Abstract. Using software (e.g., Word^®^) we split the screen and scroll through the text, finding key points that need to be in the Abstract

One method that we have used to write the Abstract is to split our computer screen (Fig. 6) and then read through our manuscript, copying key sentences from each section in the bottom half of the screen, and pasting them under the heading for the Abstract in the top half. Once we have assembled these sentences, they can then be crafted into a cohesive, brief, and engaging summary. Clearly, if you follow this advice, the Abstract will be the second to last bit of writing you do. The Title*,* which must encapsulate the entire study and lead to the Abstract, will be the last—writing backwards again!

### Revising and “reading forwards”

The need for revision should go without saying, but often it is forgotten in the haste to submit. You are telling a story. All of it must fit together (Figs. 1, 2, 4). To this end, after your paper is written—or even during the writing process—you should read it from start to finish, to see if the story works. The story must all flow, and be in the right order so the reader can fully understand it. In other words, we advocate *writing* backward, but after you do so, then *read* forward—as the reader will do—and revise your work to ensure it flows (Fig. 1C).

### You can’t polish a turd (Mackenzie 2011) and rotten wood cannot be carved (a Chinese saying)

A final note. Our advice above will be useful only if your underlying data are sound. The guidance we provide here is for writing up a study, not conducting a study. The advice must not be mistaken for guidance on experimental design or data analysis. We have assumed that your experimental design was sensible, your experiments were conducted correctly, your analysis was appropriate to address the questions you were asking, and you have arrived at logical take-home messages. In other words, we assume that there is an appropriate level of academic integrity and academic proficiency underlying your study (Table 2).

When trying to tell a good story, it may be tempting to breach these requirements. This is a mistake. Although you might present a seemingly interesting story, it would be a work of fiction, not of good science. The consequences of such behaviour can be severe. If you are caught, it is likely that an editor or reviewer will not only reject your work, but your reputation will be tarnished. Do not try to make a good story out of bad material.

## A very final note from the authors

We hope that this editorial on writing backwards provides useful guidance. If you have found it instructive, we would appreciate that you indicate this by citing our work in your Acknowledgments and including this publication in your list of references. In this way others may also see the editorial and benefit from it.
